# Stabilometric assessment of context dependent balance recovery in persons with multiple sclerosis: a randomized controlled study

**DOI:** 10.1186/1743-0003-11-100

**Published:** 2014-06-10

**Authors:** Davide Cattaneo, Johanna Jonsdottir, Alberto Regola, Roberta Carabalona

**Affiliations:** 1 Fondazione Don Gnocchi, Via Capecelatro, 66-20148 Milan, Italy

**Keywords:** Rehabilitation, Balance, Sensory strategies, Multiple sclerosis, Postural control

## Abstract

**Background:**

Balance control relies on accurate perception of visual, somatosensory and vestibular cues. Sensory flow is impaired in Multiple Sclerosis (MS) and little is known about the ability of the sensory systems to adapt after neurological lesions reducing sensory impairment. The aims of the present study were to verify whether:

1. Balance rehabilitation administered in a challenging sensory conditions would improve stability in upright posture.

2. The improvement in a treated sensory condition would transfer to a non treated sensory condition.

**Methods:**

Fifty three persons with MS, median (min-max) EDSS score of 5 (2.5-6.5), participated in a RCT and were randomly assigned to two groups. The Experimental group received balance rehabilitation aimed at improving motor and sensory strategies. The Control group received rehabilitation treatment which did not include training of sensory strategies. Persons with MS were blindly assessed by means of a stabilometric platform with eyes open, eyes closed and dome, on both firm surface and foam. Anterior-posterior and medio-lateral sway, velocity of sway and the length of Center of Pressure (CoP) trajectory were calculated in the six sensory conditions.

**Results:**

Experimental group showed statistically significant improvement (*P* < 0.05) in stability in upright posture in eyes closed condition on firm surface, and in eyes open, closed, and dome conditions on foam. No differences were observed between groups in the eyes open condition on firm surface nor in the sensory condition not addressed during the treatment.

**Conclusions:**

After rehabilitation people with MS can recover from sensory impairments thus improving upright balance. Further, the improvement seems to be context-dependent and present just in the treated sensory conditions.

**Trial registration:**

ClinicalTrials NCT02131285

## Background

Epidemiological studies have found that 25% of persons with Multiple Sclerosis (PwMS) show evidence of cerebellar and brainstem involvement already at disease onset and that this figure increases to 68% after longstanding illness [1]. Problems in these two neural systems, along with lesions in sensory systems and other neural networks, lead to frequent balance disorders, falls and fractures [2] in this population. Adequate balance relies on accurate perception of physical stimuli by visual, somatosensory and vestibular systems, and the integration of their inputs [3-5].

Therefore, difficulty in properly perceiving and integrating those stimuli can lead to inadequate motor responses [6-8]. Daley and Swank [9] assessed the postural sway in 113 PwMS, categorized either as actively ambulant but with moderate function impairment or as still ambulant but severely impaired. In quiet standing with eyes open condition 30% of the moderately impaired persons and 69% of the severely impaired persons showed abnormal body sway, whereas with eyes closed 40% of the moderately impaired persons had problems in body sway and all the severely impaired had abnormal body sway. Similarly, Cattaneo et al. [10] assessed the body sway in a sample of 53 PwMS and found that, with respect to healthy subjects, half of the people with MS showed abnormal increase of sway and velocity of sway in quiet standing with eyes open. The alteration of a single sensory input (e.g., eyes closed) led to an increase in the frequency of abnormal performances for 80% of subjects, while alteration of two sensory inputs (e.g., eyes closed on foam) led to a sharp increase in abnormal performance for almost all subjects and many of them fell during the test. Balance rehabilitation is an important component of the retraining program in people with MS [11-13]. Although the impact of MS on balance has been studied, little is known about the ability of the sensory systems to improve after neurological lesions and consequently improve balance functions and sensory integration [13]. Retraining of sensory systems is an important issue in rehabilitation and can be achieved with an approach that privileges execution of tasks in which the intact sensory systems are inhibited consequently forcing the use of the impaired sensory input. An effective improvement in sensory motor strategies would imply the transfer of skills learned in the rehabilitation setting in other, less controlled settings in which sensory information are conflicting and continuously changing. This would allow the generalization of the effect of rehabilitation observed during treatment sessions to activities of daily life. Transfer has been seen for a wide variety of motor skills in healthy persons while results in persons with neurological disabilities are controversial [14-17]. Further, little is known about transfer of sensory-motor skills learned in a specific sensory context to a different one in persons with neurological disorders. Therefore it is important to understand whether the effect of skill training in persons with MS done in specific sensory conditions transfers to other sensory conditions.

In the present paper we addressed the ability of persons with multiple sclerosis to:

1. Reduce sensory impairments improving balance. We aimed at expanding the results reported by Cattaneo et al. [18] regarding effects of balance rehabilitation on disability. Our hypothesis was that subjects repeatedly exposed to a challenging sensory condition would improve their stability with respect to a control group;

2. Transfer acquired skills among sensory conditions. We hypothesized that improvement would be context-dependent, that is, differences between groups would be detected just in sensory conditions included in the treatment regime.

## Methods

A randomized controlled study with blind assessment was set up and approved by the local Fondazione Don Gnocchi Ethics Committee. PwMS were considered eligible for the study according to the following inclusion criteria: clinically or laboratory definite relapsing-remitting, primary or secondary progressive MS, ability to stand independently in upright position for 30 seconds, ability to walk for 6 m even with an assistive device. Each participant gave written informed consent prior to the study.

### Subjects

A consecutive convenience sample of subjects with MS was assessed for eligibility and the final sample consisted of 53 subjects (Table 1). The MS subtypes were: Relapsing remitting form 45.3%; Primary progressive form 5.7%; Secondary progressive form 49%. A clinical assessment was carried out and demographical as well as clinical data were collected. EDSS (Expanded Disability Status Scale) and BBS (Berg Balance Scale) were administered [19,20]. Following this baseline assessment, subjects were assigned either to the experimental or the control group by matching the order of subjects’ entry to the hospital with a randomization list made before the start of the study. Subject and assessor were blinded with respect to group assignment.

**Table 1:** Demographic and clinical characteristics of the sample

	**EXP (N**_**EXP**_ **= 25)**	**CTRL (N**_**CTRL**_ **= 28)**	**P-Value**
Gender			
Male (%)	8 (32)	12 (42.9)	
Female (%)	17 (68)	16 (57.1)	0.1927
Age [mean (s.d.)]	48.5 (11.01)	48.2 (12.05)	0.9346
EDSS	N_EXP_ = 24		
1-4 (%)	8 (33.3)	5 (17.8)	
4.5-5.5 (%)	10 (41.7)	8 (28.6)	
6-6.5 (%)	6 (25.0)	15 (53.6)	0.1059
BBS			
≤ 44 (%)	10 (40.0)	13 (46.0)	
> 44 (%)	15 (60.0)	15 (54.0)	0.8463

EDSS: Expanded Disability Status Scale. BBS: Berg Balance Scale EXP: Experimental group; CTRL: Control group 44: cut-off score discriminating between fallers and non fallers [20].

### Stabilometric assessment

A blind assessor carried out the stabilometric assessment using the Technobody® stabilometric platform. This is a monoaxial platform, which consists of three strain gauges placed under a circular surface and sampling frequency from the transducers set at 20 Hz. Since we wanted to assess balance in conditions similar to activities of daily living we asked subjects to wear their normal comfortable shoes and clothes. The position of feet on the platform was standardized using a V shaped frame with a distance between malleolus of 3 cm. The medial border of the feet was extrarotated 12 degrees with respect to the antero-posterior axis. Subjects were tested for 30 seconds in the following six sensory conditions: eyes open, eyes closed and sway referenced, where visual enclosure was obtained by a surrounding visual device swaying consensually with subject’s body sway. The other three sensory conditions were similar but with foam pads (compliant surface) placed on the stabilometric platform under subject’s feet. Subjects were told to stay as still as possible, an operator stood behind the subject to prevent falls. Accuracy and reliability of data have been provided elsewhere [10]. Clinical and instrumental assessment at baseline was carried out in one session. Post rehabilitation, clinical and instrumental assessment was repeated and subjects were asked to use the same shoes worn during baseline assessment.

### Stabilometric variables

Instant positions of the Centre of Pressure (CoP) were computed to calculate the following variables:

•Length [mm]: length of CoP trajectory computed as sum of CoP displacement on the platform surface for each frame;

•Sway AP and ML [mm]: standard deviation of CoP time series along anterior-posterior (SwayAP) and medio-lateral (SwayML) axes;

•Velocity AP and ML [mm/s]: velocity of oscillations along anterior-posterior (VelAP) and medio-lateral (VelML) axes. These are computed as the first time derivative of CoP AP and ML displacement.

For both groups, number of subjects experiencing loss of balance during tests in the six sensory conditions pre and post rehabilitation was registered.

### Treatment

Each subject, irrespective of group assignment, had 15 treatment sessions (each lasting 45 minutes) over three weeks. Physical therapists trained for this specific study administered the rehabilitation sessions.

The *Experimental group* received balance rehabilitation to improve motor and sensory strategies, according to a task-oriented approach [3] (see Hebert et al. [13], Cattaneo et al. [18] and Additional file 1 for further details). In the experimental group, to plan treatment for sensory strategies we used the baseline results of the stabilometric assessment to identify individual subject’s sensory impairments. Subjects were given exercises in different perceptual contexts: eyes-closed condition and/or with foam pads under the feet. Exercises were carried out in the rehabilitation setting and progressed during treatment sessions from static, predictable tasks with the removal of just one sensory cue towards dynamic, unpredictable ones with the reduction of two sensory cues. Finally, exercises for improving balance during head, eyes and head-eyes movements were added [18]. In order to assess transfer of training effect from one condition to another no exercises were administered in sway referenced conditions.

The *Control group* received usual care rehabilitation comprising techniques to improve joint range of motion, muscle force, ability to perform change between different postures, and gait on firm surface [18]. Treatment for the control group was always performed with eyes open in firm surface conditions and did not include training of sensory strategies, which meant that no exercises were performed with eyes closed, on foam pad or during eyes/head movements.

Neither the Experimental nor the Control group received treatments on the stabilometric platform.

### Data analysis

Baseline differences between groups were assessed using χ^2^-test (with Yates correction where appropriate) for qualitative data (Gender, EDSS, BBS). For age, *t*-test was used after having verified of normality of distributions (Shapiro-Wilk) and homogeneity of variances (Fligner-Killen test).

In order to take into account the fact that some participants lost their balance during testing (that is, they had to grab on to hand rails to prevent falls), a transformation was done using the reciprocal of variables derived from stabilometric raw data (see Stabilometric variables section). For instance, the variable Length [mm] was transformed as 1/Lenght [1/mm]. Thus larger values imply better performances. In case of loss of balance during testing, the transformed variable was forced to zero value. Since Shapiro-Wilk test suggested a non normal distribution for transformed data, non-parametric MANOVA with 10.000 permutations and Gower distance [21] was used to assess differences between Experimental and Control group for each sensory condition. As dependent variables we considered the difference between post and pre treatment transformed values of stabilometric data. In case of statistically significant difference between groups for a given sensory condition, a post-hoc analysis was carried out using Mann–Whitney test (considering as alternative that the median shift between EXP and CTRL variables was greater than zero) with Holm correction.

Finally, we labeled as *Fallers* subjects experiencing loss of balance during testing at least in one of the six sensory conditions. McNemar test was applied to compare number of fallers pre and post treatment within groups.

Level of significance was set to 0.05. All analyses were performed using R [22] except non-parametric MANOVA, which was performed using PAST [23].

## Results

**Figure 1:**
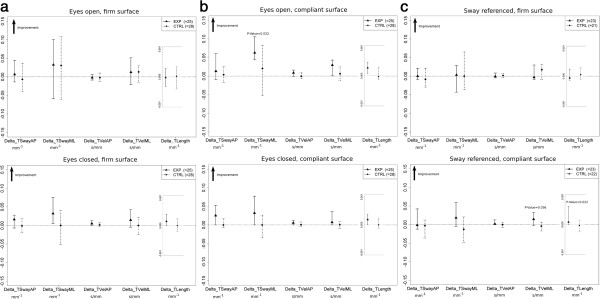
**Median and 25° − 75° percentiles of Transformed Post-Transformed Pre (i.e., Delta) values for experimental and control group. Panel a -** eyes open-firm surface and eyes closed-firm surface sensory conditions. **Panel b** - eyes open-compliant surface and eyes closed-compliant surface sensory conditions. **Panel c** - sway referenced-firm surface and sway referenced-firm surface sensory conditions. P-Values are also superimposed in correspondence to a statistically significant post-hoc comparison. EXP: Experimental Group, CTRL: Control Group. TSwayAP [expressed in mm^-1^]: reciprocal of the SwayAP of the CoP trajectory. TSwayML [expressed in mm^-1^]: reciprocal of the SwayML of the CoP trajectory. TVelAP [expressed in (mm/s)^−1^]: reciprocal of the VelAP of the CoP trajectory. TVelML [expressed in (mm/s)^−1^]: reciprocal of the VelML of the CoP trajectory. TLength [expressed in mm^-1^]: reciprocal of the length of the CoP trajectory.

**Figure 2:**
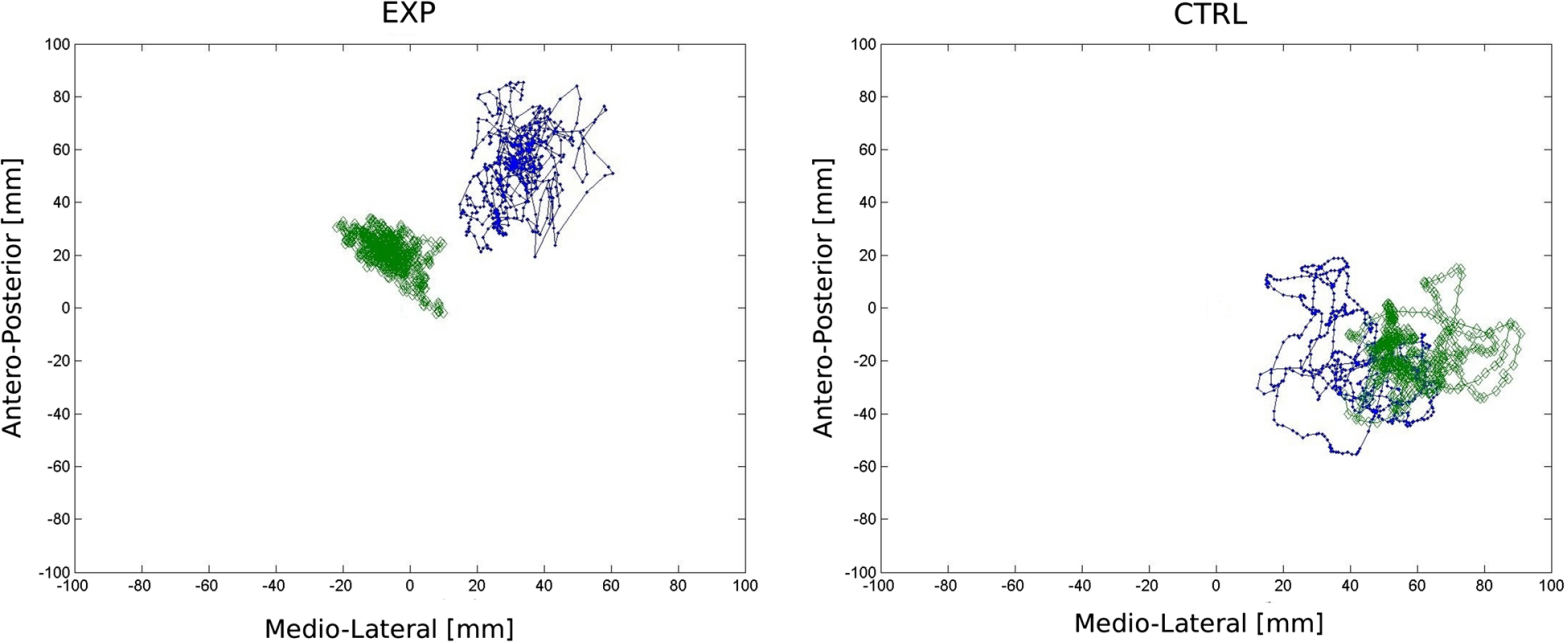
**30 seconds CoP path of a representative subject in the experimental and in the control group before and after rehabilitation.** EXP: Experimental Group, CTRL: Control Group. The center of the base of support is represented by a red asterisks; The blue lines with dots represent pre-assessment, the green lines with diamonds represent post-assessment. Data were sampled at 20 Hz.

No differences between groups were detected at baseline with respect to clinical characteristics (Table 1). With respect to the first aim of the present study, results of stabilometric assessment for transformed variables in each sensory condition are represented in Figure 1a, 1b and 1c as the difference (i.e. delta) of transformed_Post and transformed_Pre data (see also Additional file 2: Figure S1, Additional file 3: Figure S2, Additional file 4: Figure S3 for raw values). Non-parametric MANOVA found no statistically significant difference between groups for **eyes open**-**firm surface**, P-Value =0.82. A statistically significant difference between groups was found for **eyes closed**-**firm surfaces**, P-Value =0.033. In post hoc analysis none of the dependent variables reached statistical significance after Holm correction. Data for **sway referenced**-**firm surface** did not show statistically significant difference between groups (P-Value = 0.67). A statistically significant difference between groups was found for **eyes open**-**compliant surface**, P-Value = 0.01. The experimental group showed large and consistent improvements in all variables. In post hoc analysis, corrected P-Values were: 0.194 (TSwayAP), 0.032 (TSwayML), 0.069 (TVelAP), 0.069 (TVelML), 0.069 (TLength). Figure 2 reports CoP path of two representative subjects from the experimental and the control group before and after rehabilitation. Their TLength change scores (Post-Pre) were near the median change scores respectively of each group. Data for **eyes closed**-**compliant surface** showed statistically significant difference between groups (P-Value = 0.039). In post hoc analysis none of the dependent variables reached statistical significance after Holm correction. A statistically significant difference between groups were found for **sway referenced**-**compliant surface**, P-Value = 0.017. In post hoc analysis corrected P-Values were: 0.097 (TSwayAP), 0.061 (TSwayML), 0.097 (TVelAP), 0.036 (TVelML), 0.022 (TLength).

Number of subjects who lost their balance during testing (*fallers*) in the experimental and control group before and after rehabilitation are reported in Table 2. According to the McNemar test approach, we counted subjects who did lost their balance during testing before treatment and did not after treatment (label A in Table 2) and subjects who did not loose their balance during testing before treatment and did after treatment (label B in Table 2). The estimated odds ratios (A/B) were respectively 3 [Exact 95% CI 0.54-30.39] and 1 [Exact 95% CI 0.13-7.47] for the experimental and control group.

**Table 2:** Number of fallers in the experimental and control group pre and post rehabilitation

		**EXP **_**N = 23**_		**CTRL **_**N = 21**_	
		**POST**		**POST**	
		**Faller**	**Non-faller**	**Faller**	**Non-faller**
PRE	Faller	3	6_(A)_	3	3_(A)_
	Non-Faller	2_(B)_	12	3_(B)_	12

EXP: Experimental Group, CTRL: Control Group. PRE/POST: pre/post-treatment assessment.

Faller: at least one fall in one of the six sensory conditions, Non-Faller: no falls at all.

A, B: see Results section for the meaning of these labels.

## Discussion

In the present study, we hypothesized that subjects repeatedly treated in a challenging sensory condition would show an improvement in stability and that this improvement would not be present in eyes open-firm surface condition since both groups received exercises on firm surface with eyes open. We further hypothesized that improvement would be specific to trained strategies leading to a lack of improvement in sway referenced-firm surface.

Past studies showed that subjects with MS have a higher frequency of falls and balance disorders with respect to stroke [24] and elderly subjects [25] leading to injuries and deterioration of quality of life [26,27]. In spite of the fact that rehabilitation of sensory strategies is considered important for improving the ability to stand in real life [3,6] little is known about efficacy of rehabilitation in ameliorating these strategies. With respect to previous findings [18], results of the present study show that sensory strategies can be retrained in subjects with MS leading to improvement of static balance in different sensory contexts. Our results are in agreement with preliminary evidence [28,29] on the effects of rehabilitation of balance disorders in MS and corroborate results by Hebert et al. [13] on the effectiveness of rehabilitation in improving balance in MS.

According to the first hypothesis, our first aim addressed the possibility to recover from sensory impairments improving balance. The analysis of the specific sensory conditions showed no statistically significant differences between groups in **eyes open**-**firm surface**, according to the hypothesis that no difference would be expected since both groups were treated in this condition.

In contrast to what was observed by Bonan [30] on stroke patients, our data show an overall improvement in **eyes closed**-**firm surface** for EXP group suggesting that the treatment was effective in improving the use of vestibular and/or somatosensory cues and indicating a reduction in the dependence on visual information. This is an important finding because subjects with MS show deterioration of balance with eyes closed [6,7,10].

A statistically significant difference in **eyes open**-**compliant surface** was observed between the two groups suggesting a better upright balance with altered somatosensory information. This improvement could be related to the observed reduction of CoP path length that is related to energy expenditure in upright balance in the EXP group [10,31]. It has also been suggested by Prosperini [32] that path length may be a predictor of risk of falling in PwMS, so the improvement could have a potential impact on activities of daily living.

Analysis of **eyes closed**-**compliant surface** condition revealed vestibular disorders probably due to MS-related lesions in the cerebellum and brain stem leading to difficulties in the collection and integration of vestibular inputs [6]. After rehabilitation subjects in the experimental group demonstrated a statistical significant improvement in the use of this input compared to the control group. Our results suggest the possibility to improve the use of vestibular cues with balance training and are supported by the results from Hebert et al. [13] who studied the effect of 14-week vestibular rehabilitation in 38 subjects showing greater improvements in postural control in a test assessing balance in altered sensory conditions. To our knowledge, the present study is the first to demonstrate the ability of the vestibular system to adapt after rehabilitation in a specific postural condition requiring vestibular input.

A sway referenced surround was used to give conflicting information. A statistically significant difference between groups was observed in **sway referenced**-**compliant surface** (with a post-hoc statistically significant difference in TLength and TVelML), but not in **sway referenced**-**firm surface** suggesting that the improvement in balance performance seen in the experimental group may not be due to improved ability to cope with conflicting information but to better use of motor strategies: following our rehabilitation protocol, subjects understood the properties of the foam (e.g. density and viscous properties) and how to produce the necessary ankle torque to counteract body sway on this surface. Future studies may quantify changes in neuromuscular strategies used to cope with compliant surface using also electromyographic and motion analysis systems during stabilometric analysis.

A clinically strong outcome for the effectiveness of balance rehabilitation is the reduction of the number of subjects who lost balance during testing (*fallers*). We found a trend towards a status change from being a faller (before rehabilitation) to being a non faller (after rehabilitation) in the experimental group. This indicates the possibility to prevent loss of balance by means of rehabilitation of sensory strategies. Moreover, this is particularly important when considering that falling is a common problem in MS often leading to injuries, fear of falling and consequent curtailment in daily activities [33].

The second aim of the study addressed the possibility for subjects with MS to transfer the skills acquired in a specific sensory context to another not trained context. Subjects were purposely not trained in conditions with conflicting visual information (e.g. sway referenced). As hypothesized, no difference was seen between groups in condition **sway referenced**-**firm surface** suggesting that there was no transfer among sensory conditions and that effects of rehabilitation were context-specific. It has been suggested that motor learning is task dependent [15] or, in other words, that motor improvement obtained in a specific task does not transfer to other different tasks [34]. Our results suggest that this could be true also for training effects in different sensory conditions.

### Limitations of the study

A follow up would be needed to assess long term effect. Moreover, an assessment of other tasks, more related to daily living activities should be added. The large variability of individual responses to treatment should also be addressed to understand the differences among subjects and increase treatment efficacy at the individual level.

## Conclusion

In conclusion, this study suggests that persons with MS repeatedly exposed to a challenging sensory condition are able to improve the use and the integration of sensory inputs. Improvement appears to be context-dependent with no evidence of transfer among sensory conditions. Therefore, assessment in multiple sensory conditions is necessary to understand subject’s specific sensory disorder so that intervention can be aimed at improving abilities in that specific context.

## Competing interests

The authors declare that they have no competing interests. Research and research staff have been supported by the National Institutes of Health.

## Author’s contributions

DC, JJ conceived the study and collected the data. AR participated in data collection. RC conceived and managed data analysis. DC, JJ and RC completed data interpretation and prepared the manuscript. All authors read and approved the final manuscript.

## Supplementary Material

Additional file 1 **Experimental rehabilitation procedures.** Detailed description of rehabilitation protocol. Click here for file

Additional file 2: Figure S1 Eyes open-firm surface and eyes closed-firm surface stabilometric data stratified by treatment group for pre- and post-treatment assessment. Box plots report median (25–75 percentiles) values, whiskers refer to extreme values (minimum and maximum). Paired data condition is highlighted using superimposed dotplots with dots linked for each subject. Data are non transformed, thus actual sample sizes used for percentiles calculations may be lower than the nominal ones for EXP and CTRL groups according to the number of subjects who experienced loss of balance during the test (hereafter: Fallers). For each variable in each group, nominal sample sizes for EXP and CTRL as well as the number of Fallers in pre- and post-treatment assessment are reported in parentheses. Click here for file

Additional file 3: Figure S2 Eyes open-compliant surface and eyes closed-compliant surface stabilometric data stratified by treatment group for pre- and post-treatment assessment. Box plots report median (25–75 percentiles) values, whiskers refer to extreme values (minimum and maximum). Paired data condition is highlighted using superimposed dotplots with dots linked for each subject. Data are non transformed, thus actual sample sizes used for percentiles calculations may be lower than the nominal ones for EXP and CTRL groups according to the number of subjects who experienced loss of balance during the test (hereafter: Fallers). For each variable in each group, nominal sample sizes for EXP and CTRL as well as the number of Fallers in pre- and post-treatment assessment are reported in parentheses. Click here for file

Additional file 4: Figure S3 Sway referenced-firm surface and Sway referenced-compliant surface stabilometric data stratified by treatment group for pre- and post-treatment assessment. Box plots report median (25–75 percentiles) values, whiskers refer to extreme values (minimum and maximum). Paired data condition is highlighted using superimposed dotplots with dots linked for each subject. Data are non transformed, thus actual sample sizes used for percentiles calculations may be lower than the nominal ones for EXP and CTRL groups according to the number of subjects who experienced loss of balance during the test (hereafter: Fallers). For each variable in each group, nominal sample sizes for EXP and CTRL as well as the number of Fallers in pre- and post-treatment assessment are reported in parentheses. Click here for file
