# Supplementary material for: Stabilometric assessment of context dependent balance recovery in persons with multiple sclerosis: a randomized controlled study
Source: J Neuroeng Rehabil. 2014 Jun 10;11:100. doi: 10.1186/1743-0003-11-100 (PMC4065075; doi:10.1186/1743-0003-11-100)
Supplement: Additional file 1 — Experimental rehabilitation procedures. Detailed description of rehabilitation protocol. [file 1743-0003-11-100-S1.docx]

Additional file 1

Experimental rehabilitation procedures

The experimental group received balance rehabilitation to improve motor and sensory strategies during standing and walking tasks. Motor learning theory, concepts related to balance control and the task oriented approach^[[1]](#footnote-1)^ ^[[2]](#footnote-2)^were used as treatment framework.

Data from the Berg Balance Scale and subject’s reports were used to identify difficulties in the execution of daily life tasks and to detect which tasks might lead to high probability of falling. Those tasks were then used a base for treatment planning. The tasks were trained in specific sensory condition to improve sensory strategies. Based on stabilometric baseline assessment combinations of sensory conditions were chosen with respect to the individual subject’s sensory impairments. More specifically exercises aimed to force subjects in using the most impaired sensory system. That usually meant improving vestibular and somatosensory information by a reduction of visual input. For this purpose the exercises were performed in eyes-closed condition, with the use of modified lenses to reduce visual acuity and/or asking subjects to perform saccadic and pursuit eye movement to increase retinal slip and thus reducing the accuracy of visual input to detect body sway. To challenging even more the sensory system the exercises were also done with the use of foam pads under the feet to alter both visual and somatosensory information. Finally, tasks for improving balance during head, eyes and head and eyes movements were added.

During the execution of the identified tasks we paid attention to postural alignment, especially to the attitude of axial segments. More attention was directed toward the patient’s ability to detect the position and movements of the centre of mass and to control them. The ability to explore limits of stability with a voluntary shift of the centre of mass was also requested. Axial and postural anticipatory strategies were improved using reaching tasks and the manipulation of object with different sizes and weights. During gait activities two main aspects were addressed: abnormal movement of the centre of mass, especially in the frontal plane, and eyes-head and trunk stability. The exercises generally progressed from static tasks toward exercises carried out during gait activities. Modelling technique and verbal feedback from therapists were used as feedback cues. The generalization of results were obtained introducing dual tasks exercises.

1. Bayona NA, Bitensky J, Salter K, Teasell R. The role of task-specific training in rehabilitation therapies. Top Stroke Rehabil. 2005;12:58-65
    [↑](#footnote-ref-1)
2. Carr JH, Shepherd RB. A Motor Relearning Program for Stroke, 2nd ed. Rockville, MD: Aspen Publishers; 1987. [↑](#footnote-ref-2)
