# Supplementary figures and images for: Stabilometric assessment of context dependent balance recovery in persons with multiple sclerosis: a randomized controlled study
Source: J Neuroeng Rehabil. 2014 Jun 10;11:100. doi: 10.1186/1743-0003-11-100 (PMC4065075; doi:10.1186/1743-0003-11-100)

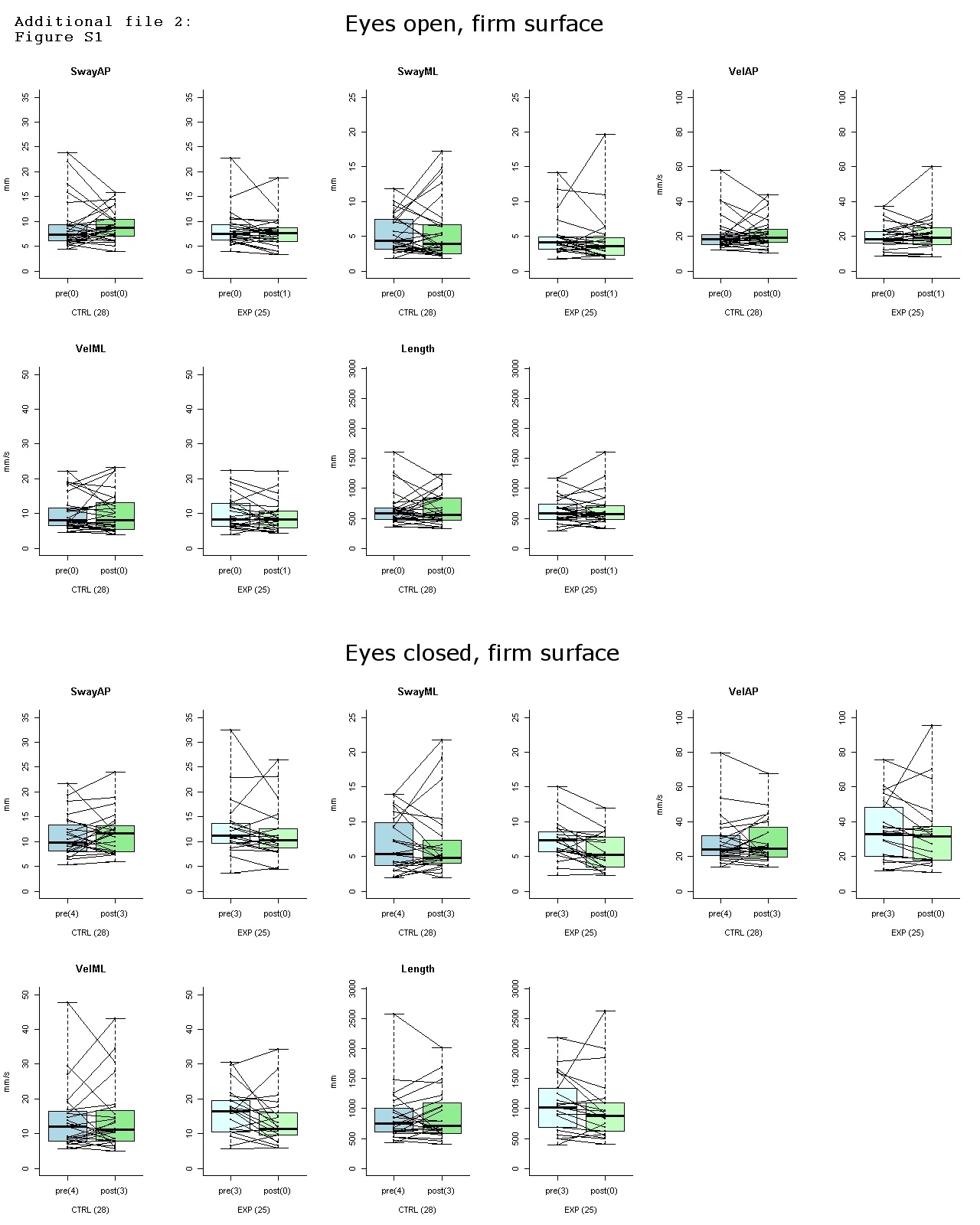

Supplement: Additional file 2: Figure S1 — Eyes open-firm surface and eyes closed-firm surface stabilometric data stratified by treatment group for pre- and post-treatment assessment. Box plots report median (25–75 percentiles) values, whiskers refer to extreme values (minimum and maximum). Paired data condition is highlighted using superimposed dotplots with dots linked for each subject. Data are non transformed, thus actual sample sizes used for percentiles calculations may be lower than the nominal ones for EXP and CTRL groups according to the number of subjects who experienced loss of balance during the test (hereafter: Fallers). For each variable in each group, nominal sample sizes for EXP and CTRL as well as the number of Fallers in pre- and post-treatment assessment are reported in parentheses. [file 1743-0003-11-100-S2.jpg]

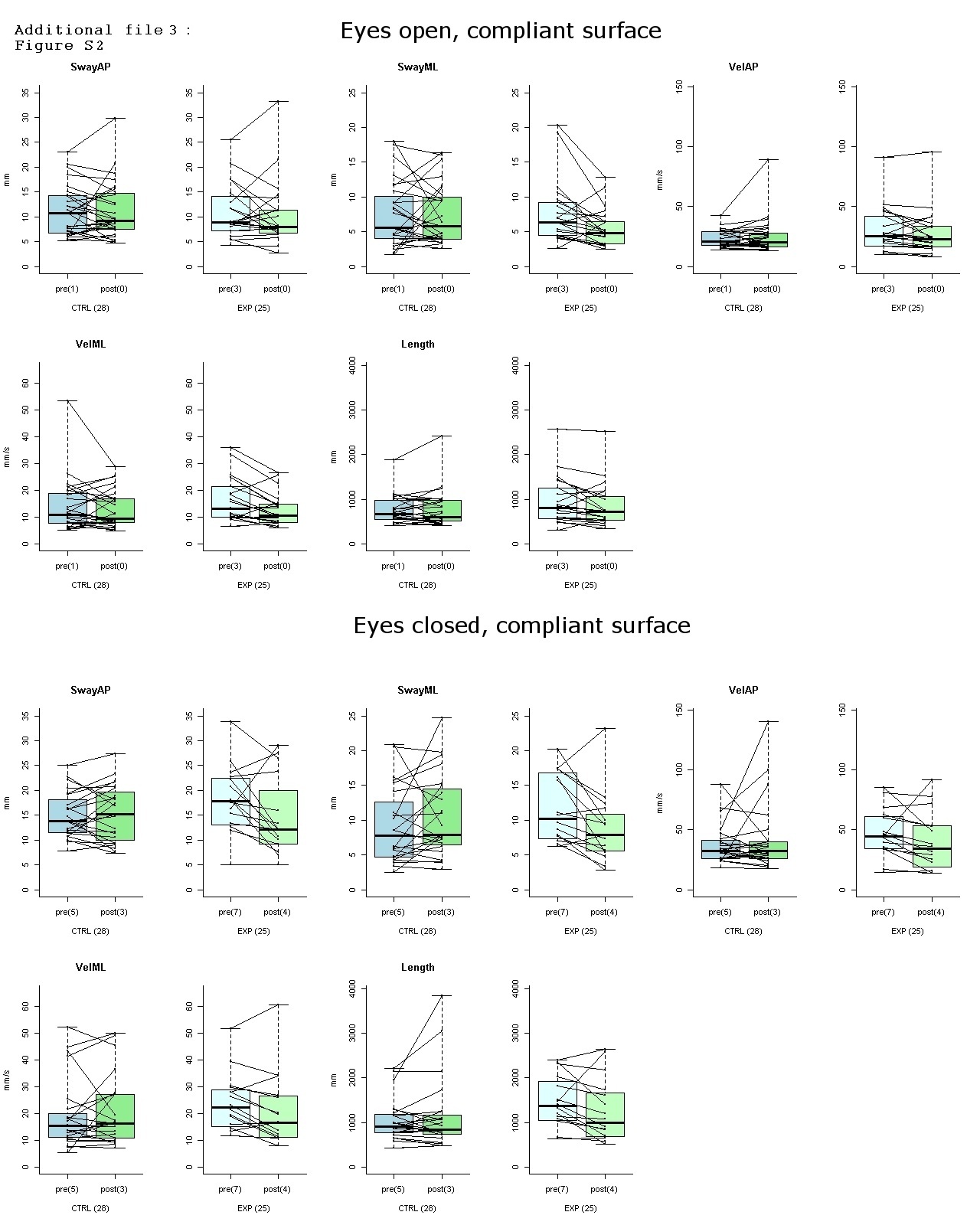

Supplement: Additional file 3: Figure S2 — Eyes open-compliant surface and eyes closed-compliant surface stabilometric data stratified by treatment group for pre- and post-treatment assessment. Box plots report median (25–75 percentiles) values, whiskers refer to extreme values (minimum and maximum). Paired data condition is highlighted using superimposed dotplots with dots linked for each subject. Data are non transformed, thus actual sample sizes used for percentiles calculations may be lower than the nominal ones for EXP and CTRL groups according to the number of subjects who experienced loss of balance during the test (hereafter: Fallers). For each variable in each group, nominal sample sizes for EXP and CTRL as well as the number of Fallers in pre- and post-treatment assessment are reported in parentheses. [file 1743-0003-11-100-S3.jpg]

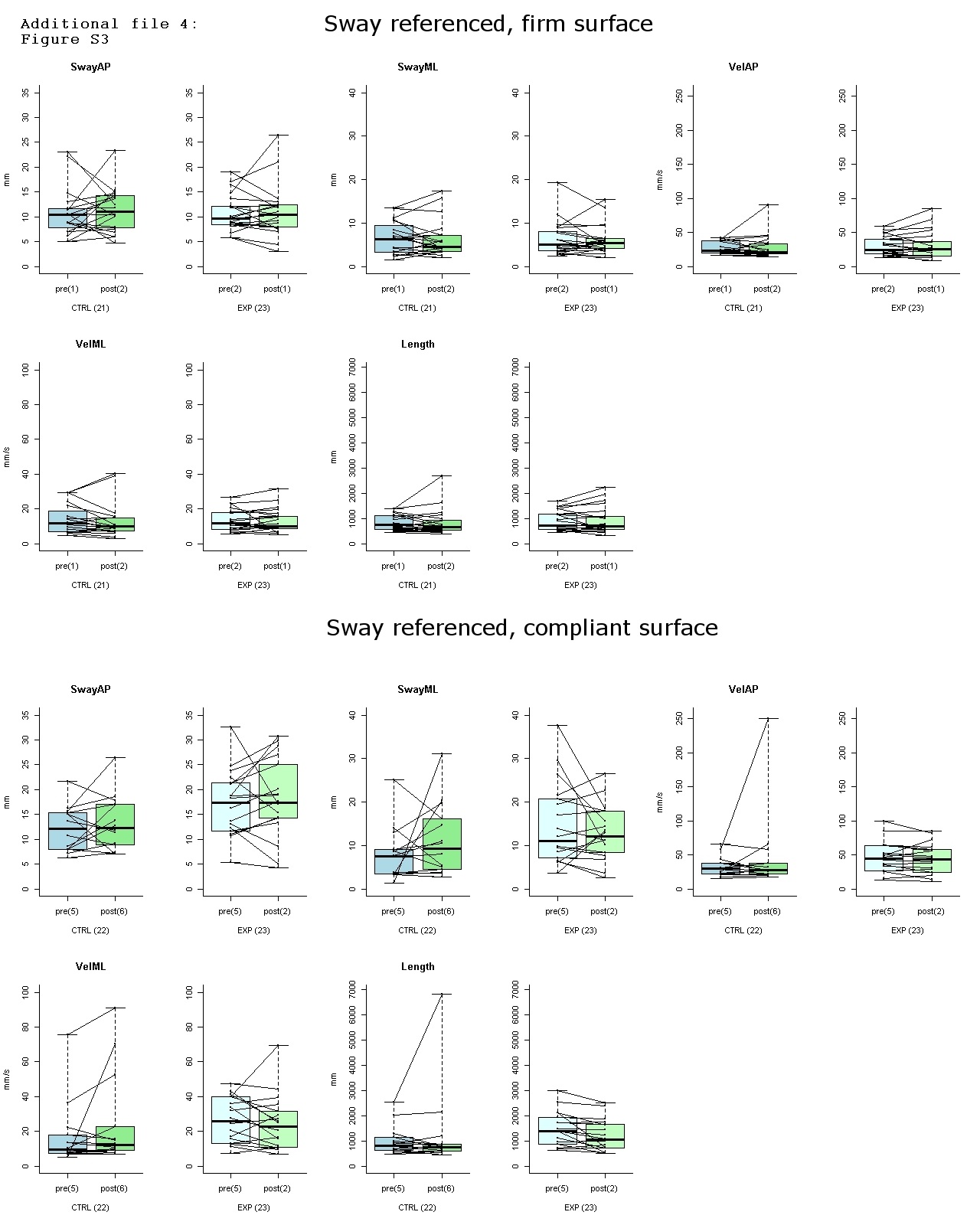

Supplement: Additional file 4: Figure S3 — Sway referenced-firm surface and Sway referenced-compliant surface stabilometric data stratified by treatment group for pre- and post-treatment assessment. Box plots report median (25–75 percentiles) values, whiskers refer to extreme values (minimum and maximum). Paired data condition is highlighted using superimposed dotplots with dots linked for each subject. Data are non transformed, thus actual sample sizes used for percentiles calculations may be lower than the nominal ones for EXP and CTRL groups according to the number of subjects who experienced loss of balance during the test (hereafter: Fallers). For each variable in each group, nominal sample sizes for EXP and CTRL as well as the number of Fallers in pre- and post-treatment assessment are reported in parentheses. [file 1743-0003-11-100-S4.jpg]
